# The gap between clinical gaze and systematic assessment of movement disorders after stroke

**DOI:** 10.1186/1743-0003-9-61

**Published:** 2012-08-27

**Authors:** Hanneke JM van der Krogt, Carel GM Meskers, Jurriaan H de Groot, Asbjørn Klomp, J Hans Arendzen

**Affiliations:** 1 Department of Rehabilitation Medicine, Leiden University Medical Centre, Leiden, Netherlands; 2 Faculty of Mechanical, Maritime and Materials Engineering, Delft University of Technology, Delft, Netherlands

**Keywords:** Stroke, Biomechanics, Electromyography, Outcome measures

## Abstract

**Background:**

Movement disorders after stroke are still captured by clinical gaze and translated to ordinal scores of low resolution. There is a clear need for objective quantification, with outcome measures related to pathophysiological background. Neural and non-neural contributors to joint behavior should be separated using different measurement conditions (tasks) and standardized input signals (force, position and velocity).

**Methods:**

We reviewed recent literature for the application of biomechanical and/or elektromyographical (EMG) outcome measures under various measurement conditions in clinical research.

**Results:**

Since 2005, 36 articles described the use of biomechanical and/or EMG outcome measures to quantify post-stroke movement disorder. Nineteen of the articles strived to separate neural and non-neural components. Only 6 of the articles measured biomechanical and EMG outcome measures simultaneously, while applying active and passive tasks and multiple velocities.

**Conclusion:**

The distinction between neural and non-neural components to separately assess paresis, stiffness and muscle overactivity is not commonplace yet, while a large gap is to be bridged to attain reproducible and comparable results. Pathophysiologically clear concepts, substantiated with a comprehensive and concise measuring protocol will help professionals to identify and treat limiting factors in movement capabilities of post-stroke patients.

## Review

### Introduction

Movement disorders after stroke are the result of a complex interaction of primary neural damage and secondary tendomuscular changes [1,2]. The combination of paresis, stiffness and muscle overactivity leads to a phenotype that is easy to recognize clinically, but hard to quantify [1]. The broadly used term “spasticity” is under debate. Different definitions are used, and while it is mostly used as an umbrella-term for the phenotype, it describes only a part of the movement disorder [3-7], and has little relation to the capabilities of a patient to perform under different circumstances.

Clinical gaze and manual tests to assess movement disorder after stroke are readily available to every physician and are currently used as a basis for clinical practice. However, there are some difficulties in evaluating interventions within patients and between studies. For example, resolution of clinical tests is low, rater dependency is variable and conditions are difficult to standardize [8,9]. Little is known about responsiveness of the clinical tests to change. Ordinal scales are often misused as linear entities. Also, the measured construct of tests is not always taken into account when choosing a test for the assessment of stroke patients [9], i.e. improvement in tests on the domains of body structures and functions of the International Classification of Functioning, Disability and Health (ICF) do not automatically lead to improvement in the domains of activities and participation.

Correct use of a meaningful pathophysiological construct will enable clinicians to target their expensive and labor intensive therapies such as botulinum toxin and exercise programs more efficiently and effectively. Evidently, this challenges the community of rehabilitation specialists to quantify and objectify the components of movement disorders according to their pathophysiological origin [10,11] and their relevance for performance in the different ICF domains. For the domain of Body Structures and Body Functions this means that, first of all, input signals (e.g. velocity, force, angle) should be standardized to enable comparability and repeatability. Second, multiple measuring conditions should be applied to trigger the different pathophysiological components [10,11], i.e. active tasks to study voluntary muscle properties, passive tasks to study passive tissue properties, and multiple measurement velocities to elicit stretch reflexive behavior. This will allow for differentiation in neural and non-neural components (see Table 1), and will enable clinicians to direct their therapies more precisely. Simultaneously used biomechanical and electrophysiological techniques can support the identification of active, passive and reflexive components and their complex (non linear) interactions.

**Table 1:** Division of components of post stroke movement disorder in non-neural and neural properties offers a construct for targeted therapy: an overview

	***Measuring condition***	***Construct***
Non-neural	Passive	Stiffness, changed properties of connective tissue and joints
Neural	Active	Paresis, diminished voluntary muscular capacity
	Reflexive (velocities)	Muscle overactivity, stretch reflex behavior

Recommendations for objective and quantitative assessment of movement disorders after stroke are readily available [6,9,12,13]. However, it is unclear to which extent these recommendations are implemented in current research and clinical practice. The aim of the present paper is to provide an overview of biomechanical and electrophysiological outcome measures recently used to describe post-stroke movement disorders. In addition, the use of underlying pathophysiological constructs is investigated.

### Methods

**Figure 1:**
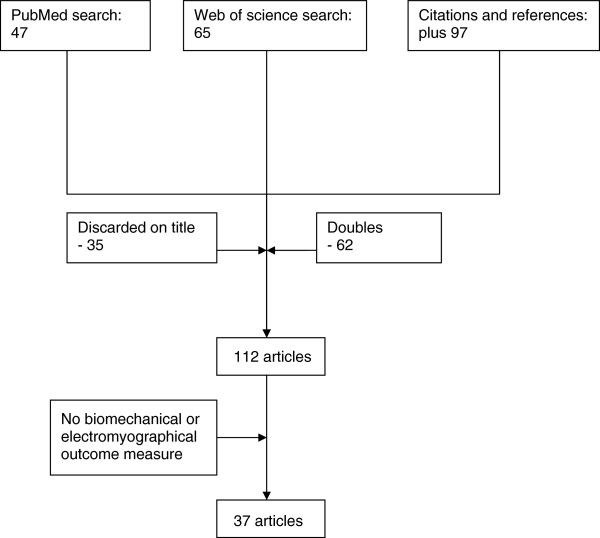
A flow chart of the search strategy and outcome.

We conducted a literature search on PubMed and Web of Science with the following search terms: PubMed: stroke AND biomechanics AND electromyography (limits: last 5 year, human, adult)(accessed dec 2010). Web of Science: TS = ((stroke AND outcome measures) AND (biomechanic OR electromyography)). We also tracked references and citations. Thereafter we checked for doubles and scanned titles and abstracts. For a flow chart of the search, see Figure 1.

Within the found references, we identified biomechanical and electromyographical (EMG) outcome measures, used in research on stroke patients. We searched for the pathophysiological construct of these outcome measures, given by the authors. Biomechanical and EMG outcome measures were examined for task instruction (active or passive) and for applied velocities of perturbations (slow, fast or multiple velocities). Subsequently, the outcome measures were separated in clusters, according to the applied method. For biomechanical outcome measures, the clusters were: range of motion, stiffness (or resistance to passive movement), maximum voluntary contraction, viscosity, work, mathematical models, other. For EMG outcome measures, the clusters were: magnitude, threshold (angle), onset (time), co-activation, other.

### Results

The search yielded 37 articles. A flowchart of the search is illustrated in Figure 1. Study characteristics (measured segment, number of subjects, category of research) and the biomechanical and electrophysiological outcome measures found in each article, are summarized in Additional file 1: Table S1.

**Figure 2:**
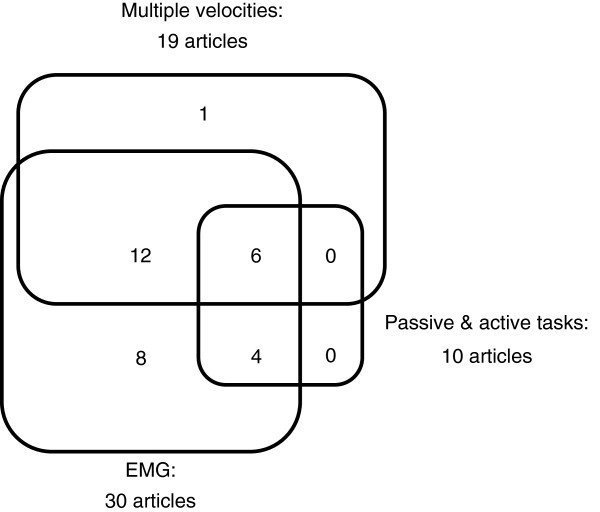
Number of articles conforming to recommendations for measuring movement disorder after stroke: measuring active and passive tasks, measuring at multiple velocities and including EMG-techniques.

Of the 37 articles, 3 were review articles [14-16]. In the other 34 articles, 30 included EMG outcome measures [17-45] and 31 included biomechanical outcome measures, while 25 articles included both. Active and passive tasks were found in 10 articles [17,19,20,26,27,29,33,34,38,46]. Different measuring velocities were found in 19 articles [18-22,24,27,30-35,37-39,43,44,47]. In 6 articles, all of the aforementioned properties were present (see Figure 2) [19,20,27,33,34,38].

In 6 articles the biomechanical and/or EMG were used to evaluate treatment of stroke patients [17,26,28,31,37,43], 10 articles addressed reliability or feasibility of the outcome measures in stroke patients [20,21,23,32,35,44,45,47-49] and 18 articles were observational (difference between healthy subjects and stroke patients) or tested a new measuring method [18,19,22,24,25,27,29,30,33,34,36,38-42,46,50]. A total of 682 stroke patients and 175 healthy subjects were included (see Additional file 1: Table S1).

In 25 articles the biomechanical and EMG techniques were used to objectify or quantify a clinical concept (e.g. spasticity, muscle tone, muscle activity, impairment or coupling) [18,20,22-27,29-33,35,37,39-47,50]. Alongside this, a large part of the articles use these techniques to separate the underlying (neural and non-neural) mechanisms of the concept (n = 19) [18-24,30,31,33-35,37,39,40,42-45]. Finally, there is also a small number of articles that advocate standardized input (n = 6) [19-21,24,30,40].

#### Biomechanical outcome measures

An overview of biomechanical outcome measures is presented in Additional file 1: Table S1.

Range of motion was assessed as passive range of motion (pain-free or comfortable range of movement about a joint) (n = 12), active range of motion (n = 3) or both (n = 3). An electrogoniometer was used in 7 articles [17,18,22,25,35,46-48], customized devices were used in 8 articles [19,20,32,34,38,44,45,49] and in 2 articles manual goniometry was used to measure the range of motion [23,28].

Maximum voluntary contraction was measured with a handheld dynamometer (n = 1) [17], or a torque transducer/load cell in a (customized) device (n = 11) [19,27-29,33,34,36,38,39,41,49]. Isometric conditions were applied in 11 articles, while in 1 article the peak active torque during flexion/extension movement was measured [19].

Stiffness or resistance to passive movement was measured as force or torque versus angle during passive movement, with the identical device as used for maximum voluntary contraction. The methods ranged from measuring peak resistance during movement (n = 2) [30,48], calculating the slope of the force-angle curve, linearized over a part [19,20,22,23,32,43] or the total [24,30,32,35,43] of the movement trajectory (n = 10), to a model fit (n = 5) [33,34,38,44,45]. A minority compared stiffness at different velocities (n = 5) [24,30,32,35,43].

Viscosity (n = 3) was derived from force and position at different velocities during passive movement [29,31,37]. Work (n = 2) was calculated as the area under the curve of moment-angle, during passive movement [18,30]. Mathematical models (n = 3) were used to compare the estimated or predicted parameter with the actual parameter. This was done once for muscle length [27], once for torque [33] and once for angle trajectory [42].

Other biomechanical parameters assessed (n = 25) were tracking index (correlation between target angle and actual angle) [19,20], relaxation index (difference in angle between initial angle and first drop in pendulum test) [25], velocity dependent torque [18,33-35,44,45], phase dependent torque (timing of joint resistance compared to movement) [29,37], movement pattern (range of motion related to duration of phase) [46], miscellaneous other torque parameters [18,23,30,38,40,50] and gains [44,45]. Most attempted to cipher some spasticity parameter, using different combinations of velocities or positions and resulting torque.

#### Electrophysiological outcome measures

All electrophysiological outcome measures were measured using surface electromyography (EMG). An overview of EMG outcome measures is presented in Additional file 1: Table S1.

Magnitude of EMG signal was measured during maximum voluntary contraction (isometric) (n = 5) [27,28,33,39,41], with a target force or target EMG-level (n = 2) [27,39], during passive movement (n = 18) [17-20,22-26,30,33,36,38,40,43,44,46] or during active movement [46]. Tendon taps were used in 2 articles [23,40] and H-reflex stimulation was used in 2 articles [26,36]. In all cases the EMG was rectified and/or normalized. The EMG activity during maximum voluntary contraction was mostly used as a reference value for the magnitude of reflex EMG response. In 6 cases, EMG activation was compared between different velocities or task instructions [22,30,35,39,43,46].

Threshold was described as the angle at which EMG activity started during passive movement. Thresholds were compared between different velocities of perturbation [21,30,31,37]. Onset was described as the latency in time between start of perturbation and start of EMG activity [27,33,34,38,45].

Co-activation (or cocontraction) compared agonistic and antagonistic EMG-activity during passive movement (n = 4) [19,20,44,45], during active movement (n = 1) [46] or maximum voluntary contraction (n = 1) [34].

Other parameters of EMG (n = 9) that were assessed, include velocity dependent EMG signal [35], tonic threshold (extrapolation of thresholds from different velocities to zero velocity) [21], duration of activity [29], modulation of activity [29], volitional response time [27,39], slope of recruitment curve and H-reflex related parameters [36].

#### Pathophysiological construct of outcome measures

Observed pathophysiological constructs were spasticity (n = 16) [17,21-26,32,33,35,37,44-48], muscle tone [18,30,31,42,43], muscle overactivity [28,39,40,50], paresis [49], motor control [29], impairment [19,20], coupling between extremities [27,36,38], secondary changes [34] and normalization of signals [41]. Observed underlying mechanisms used to underpin the pathophysiological constructs were paresis [17,19-21,24,34,49], limited range of motion [19,20,46,50], stiffness/hypertonia [18-25,30-35,37,38,42-45,47,50], muscle overactivity/hyperreflexia [17,20-25,27-31,34-40,43,45,50] and motor control/dexterity [19,20,29,33,46,49]. An overview of the cross-links between observed pathophysiological constructs and underlying mechanisms is presented in Table 2.

**Table 2:** Concepts and pathophysiological mechanisms categorized in articles measuring movement disorder after stroke

***Concept***	***Articles (n)***	***Pathophysiological mechanisms***				
		**Paresis**	**Limited range of motion**	**Stiffness/ hypertonia**	**Muscle overactivity/ hyperreflexia**	**Motor control/ dexterity**
Spasticity	16	3	2	12	9	2
Muscle tone or hypertonia	5	0	0	5	3	0
Muscle overactivity	4*	0	1	1	4	0
Other						
- paresis	1	1	0	0	0	1
- motor control	1	0	0	0	1	1
- impairment	2	2	2	2	1	2
- coupling	3^#^	0	0	1	3	0
- secondary changes	1	1	0	1	1	0
- normalization	1	0	0	0	0	0

*muscle overactivity (n = 2), reflex response (n = 2); ^#^ affected & non affected side (n = 1), upper & lower extremity (n = 1), proximal & distal segment of extremity (n = 1).

The most addressed concept was that of spasticity (n = 16), although different definitions and interpretations were given [17,21-26,32,33,35,37,44-48]. The observed underlying mechanism was in either non-neural (stiffness, resistance) (n = 4) [32,33,45,47], neural (muscle overactivity, hyperreflexia) (n = 2) [17,46], or a combination (n = 8) [21-25,35,37,45]. The remaining articles concerning the concept of spasticity did not discriminate between neural and non-neural components (n = 2) [26,48].

The second most addressed concept was that of muscle tone (n = 5) [18,30,31,42,43]. All five use non-neural components of muscle tone as underlying mechanism (i.e. stiffness, inertia, mechanical characteristics of passive tendomuscular and connective tissue, mechanical characteristics of activated muscle), while neural components (muscle overactivity, hyperreflexia) were separately addressed in 3 articles [30,31,43].

The concept of muscle overactivity was the main topic in 4 articles [28,39,40,50]. Two articles distinguish between neural and non-neural properties (n = 2) [39,40].

The underlying mechanisms of stiffness and muscle overactivity were combined in 15 out of the 37 articles [19,21-25,30,31,34,35,37,38,40,43,45].

### Discussion

Since 2005, 37 articles described the use of biomechanical and/or EMG outcome measures to describe post-stroke movement disorder. Nineteen of the articles strived to separate neural from non-neural components. The most frequent pathophysiological constructs were spasticity, muscle tone and muscle overactivity. Only 6 of the articles measure biomechanical and EMG outcome measures simultaneously, while applying active and passive tasks and multiple velocities.

Whilst this study limited the use of search engines to PubMed and Web of Science, it is likely that the main bulk of relevant literature is identified by using generic search terms and cross-checking references. The restriction to search only recent literature is justified by the specific aim of the study, namely, to identify current methods.

This review shows that in recent years initiatives have been taken to quantify and objectify measurements in post stroke movement disorders. It also indicates that the conceptual mainframe of separating movement disorder into neural and non-neural components was not always taken into account, i.e. active, passive and reflex contributions were not always divided. In some articles, there was a lack of consistency in administration of the underlying pathophysiological mechanism (paresis, increased stiffness and muscle overactivity) or pathophysiological concept (spasticity, muscle tone). For example: one [50] of the 4 papers on muscle overactivity did not use EMG. Another example is spasticity, which was described as velocity dependent in 13 of the 16 papers [17,21,23-26,32,33,35,44,45,47,48], while only 8 of the 16 papers use multiple velocities in their tests [21,22,24,32,33,35,37,47].

Measuring in different operating points is not commonplace yet, while it will allow for a more complete understanding of the capabilities of a patient with a movement disorder. Active and passive tasks instructions will give information about paresis and involuntary muscle activity, and a variation of velocities of perturbations will illuminate stiffness and reflex contributions. A more specific knowledge of the capabilities of a patient will probably lead to a more specific treatment. For example, patients with movement disorder due to severe paresis or reduced range of motion through secondary changes will not benefit from spasmolytic or neurolytic treatment. However, before treatment in spastic patients, these disorders are not systematically separated from muscle overactivity. This does not benefit the individual patient, is not cost effective and will introduce a bias in research of effect measurements after treatment.

The techniques as described in this review are mostly not available in clinical practice yet. This has led to prolonged use of clinical scores, despite their known disadvantages. We recommend that future work on movement disorder in stroke patients should be based on a clear concept and include a comprehensive and concise measurement protocol which is easily applied on and well tolerated by stroke patients. Outcome measures should be pathophysiologically meaningful and applicable in decision making for clinicians. Additionally, to increase the understanding of primary and secondary changes, longitudinal studies will be essential [51]. This will enable specialists in physical medicine and rehabilitation to tailor their therapies and, moreover, allow them to assess the effect of (experimental) interventions.

### Conclusion

In the last 6 years a number of initiatives were developed to quantify and objectify movement disorder after stroke. However, the distinction between non-neural and neural components to separately assess paresis, stiffness and muscle overactivity, is not commonplace yet. A large gap has to be bridged to attain reproducible and comparable results.

Pathophysiologically clear concepts, substantiated with a comprehensive and concise measuring protocol will help professionals to identify and treat limiting factors in movement capabilities of post-stroke patients.

## Abbreviations

EMG: Electromyography; H-reflex: Hoffmann reflex, EMG response of muscle after electrical stimulation of the afferent nerve fibers; EXPLICIT-stroke: “EXplaining PLastICITy after stroke”; mAS: Modified Ashworth Score; ICF: The International Classification of Functioning, Disability and Health is a classification of health and health-related domains. These domains are classified from body, individual and societal perspectives by means of two lists: a list of body functions and structure, and a list of domains of activity and participation. Since an individual’s functioning and disability occurs in a context, the ICF also includes a list of environmental factors [ http://www.who.int accessed May 6th 2011].

## Competing interests

The authors declare that they have no competing interests.

## Authors’ contributions

JMK contributed to the design of the study, carried out the literature search and wrote the manuscript. CGM is co-PI of the EXPLICIT study, contributed to the design of the present study, assisted in data interpretation and commented on the manuscript. JDG contributed to the design of the study, assisted in data interpretation and commented on the manuscript. AK contributed to the design of the study, assisted in data interpretation and commented on the manuscript. JHA is co-PI of the EXPLICIT study, contributed to the design of the study, assisted in data interpretation and commented on the manuscript. All authors read and approved of the manuscript.

## Supplementary Material

Additional file 1 **Table S1.** Study characteristics (measured segment, number of subjects, category of research) and the biomechanical and electrophysiological outcome measures found in each article. Click here for file
